# Prostaglandin E2 Labour Induction with Intravaginal (Minprostin) versus Intracervical (Prepidil) Administration at Term: Randomized Study of Maternal and Neonatal Outcome and Patient's Perception Using the Osgood Semantic Differential Scales

**DOI:** 10.1155/2014/682919

**Published:** 2014-12-29

**Authors:** Joscha Reinhard, Roberta Rösler, Juping Yuan, Sven Schiermeier, Eva Herrmann, Michael H. Eichbaum, Frank Louwen

**Affiliations:** ^1^St. Marienkrankenhaus, Richard-Wagner-Straße 14, 60318 Frankfurt am Main, Germany; ^2^Department of Obstetrics and Gynaecology, Faculty of Medicine, Johann Wolfgang Goethe University Frankfurt am Main, Theodor-Stern-Kai 7, 60590 Frankfurt am Main, Germany; ^3^Marien-Hospital Witten, Marienplatz 2, 58452 Witten, Germany; ^4^Institute of Biostatistics and Mathematical Modelling, Johann Wolfgang Goethe University Frankfurt am Main, Theodor-Stern-Kai 7, 60590 Frankfurt am Main, Germany

## Abstract

*Aim*. To compare the efficacy, safety, and patient's perception of two prostaglandin E2 application methods for induction of labour. *Method*. Above 36th weeks of gestation, all women, who were admitted to hospital for induction of labour, were prospectively randomised to intravaginal 1 mg or intracervical 0.5 mg irrespective of cervical Bishop score. The main outcome variables were induction-to-delivery interval, number of foetal blood samples, PDA rate, rate of oxytocin augmentation, rate of vaginal delivery, and patient's perception using semantic differential questionnaire. *Results*. Thirty-nine patients were enrolled in this study. There was no statistical significant difference between the two groups in regard to perceptions of induction. The median induction delivery time using intravaginal versus intracervical administration was 29.9 versus 12.8 hours, respectively (*P* = 0.04). No statistically difference between the groups was detected in regard to parity, gestation age, cervical Bishop score, number of foetal blood samples, PDA rate, rate of oxytocin augmentation, and mode of birth. *Summary*. Irrespective of the cervical Bishop Score, intracervical gel had a shorter induction delivery time without impingement on the women's perception of induction.

## 1. Introduction

In order to reduce the risk of maternal or neonatal morbidity and mortality, labour is often induced [1]. Approximately one in four or five women in Europe and USA is induced due to prolonged pregnancy, prelabour rupture of the membranes, and concerns about the well-being of the child or mother (e.g., poor growth, cholestasis, preeclampsia, etc.) [2]. Prostaglandins have been used for induction of labour since the 1960s [2, 3] and are widely used in clinical practice, but side-effects are reported including gastrointestinal symptoms (nausea, diarrhoea, and vomiting), uterine hyper stimulation, and fever [2, 4]. Various prostaglandin preparations are available, which have been used by various routes, including local (intracervical and intravaginal) and general administration (oral, intramuscular, and intravenous). For local administration, prostaglandin E2 (PGE2) is available in a gel in general 0.5 mg for intracervical use and 1 or 2 mg for intravaginal use. However, various other dosages and sustained-release pessary are also currently used [2, 3]. In many centres, the prostaglandin E1 (PGE1) analogue misoprostol has replaced the use of PGE2 analogue for induction of labour; however, in Germany, this is still an “off-label” use with legal implications and PGE1 are not used in our hospital.

This study was carried out to examine whether the intracervical versus intravaginal PGE2 administration causes more discomfort to the woman. In various studies, prostaglandin was applied intracervically during a vaginal examination; however, especially for an unripe cervix, this is a very difficult procedure to perform [5]. Therefore, in this study, all intracervical PGE2 applications were applied identifying the cervix during a speculum examination and the insertion of a cannula in the cervix while viewing the cervix. Secondary study objectives were the labour induction time and maternal as well as foetal outcome.

## 2. Materials and Methods

All women, who were admitted to hospital for labour induction with a singleton foetus in vertex presentation, at or above 36 + 1 gestation age and absence of active labour, were enrolled for this study from March 2013 to August 2013. Exclusion criteria were multiple pregnancies and previous uterine surgery (e.g., caesarean section and myomectomy). The patient's baseline characteristics and reasons for induction are presented in Table 1. All patients gave their informed consent and were prospectively randomised using the web based programme “random” (http://www.random.org/integers/ 1 = intravaginally PGE2 and 2 = intracervically PGE2) irrespective of the cervical Bishop score.

The Osgood semantic differential score [6–8] is a validated questionnaire, which has been modified by Ertel and can be used to quantify emotional changes [9–11]. On a list of bipolar scales, the participants had to score from −4 to +4 without a middle or neutral point. Before the first induction and after birth, the Osgood semantic differential questionnaire was evaluated for the word “induction.”

All patients in the intravaginal PGE2 group received first 1 mg (Minprostin©) and in the absence of regular contraction another 2 mg was applied after 6 to 8 hours. On the 2nd day of induction, initially 2 mg and after 6 to 8 hours another 1 mg were applied if no regular contraction was felt by the patient.

All patients in the intracervical PGE2 group were placed in the lithotomy position and a speculum examination was done to identify the cervix. The insertion of a cannula in the cervix and application of the 0.5 mg gel (Pepidil©) were carried out whilst viewing the cervix. In the absence of regular contraction, another 0.5 mg gel was applied into the cervix using the same procedure.

All patients had fetal electrocardiography and electrohysterogram (Monica AN24) controls half an hour before gel application and one hour after gel application whilst lying on the right or left side. No further dose was given if contractions exceeded 2 per 10 minutes. After three days of failed induction, a switch of application method was allowed. In the presence of >2 contractions per 10 minutes, failure of cervical dilatation (<1 cm/h) or failure to progress during the active first (cervix dilatation >3 cm) and second stages of labour, management required initiation of intravenous oxytocin by infusion was started >6 h after the last dose of gel. Electrocardiotocography was intermittent during active phase of labour (cervical dilatation >3 cm) and continuous if in the active phase of labour the foetal heart rate was suspect or pathological. During 2nd stage of labour, continuous foetal monitoring was carried out.

Outcome parameters were the change in women's semantic differential questionnaire response after birth subtracted before initiation of induction of labour. Secondary outcome parameters were the induction-to-delivery interval (from insertion of first gel to birth), number of foetal blood samples, PDA rate, rate of oxytocin augmentation, 5 min and 10 min Apgar score, and arterial pH value.

The data analysis used SPSS (Version 21, IBM© SPSS© Statistics). Differences between groups were tested by a nonparametric Mann-Whitney test. Two-sided *P* values were reported for all tests and a value < 0.05 was regarded as significant.

Ethical approval was given by the local ethics committee.

## 3. Results

**Figure 1:**
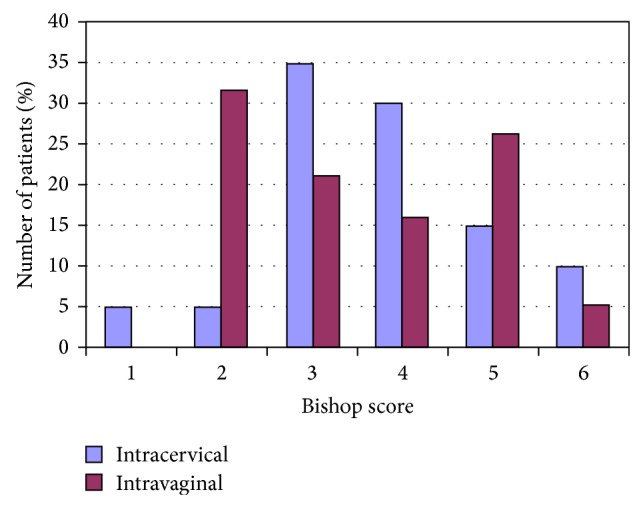
Bishop score at admission to hospital.

**Figure 2:**
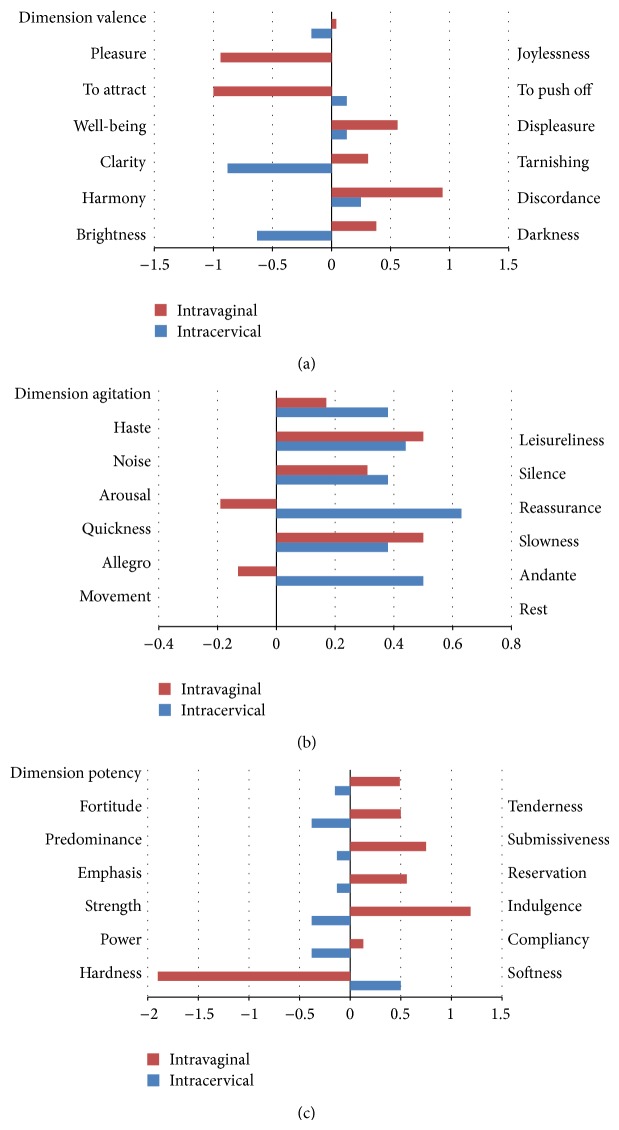
Semantic differential score after birth subtracted with the score before initiation of induction: (a) dimension valence [average of all separate items; negative value → “positive change” → pleasure, to attract, well-being, clarity, harmony, brightness; positive value → “negative change” → joylessness, to push off, displeasure, tarnishing, discordance, darkness], (b) dimension agitation [average of all separate items; positive value → leisureliness, silence, reassurance, slowness, andante, rest], and (c) dimension potency [average of all separate items; negative value → fortitude, predominance, emphasis, strength, power, hardness; positive value → tenderness, submissiveness, reservation, indulgence, compliancy, softness].

Thirty-nine patients were enrolled in this study. Nineteen patients were randomised in the vaginal group and 20 patients in the cervical group. There were no differences between the two groups in respect to maternal age, gravity, parity, gestational diabetes, previous conization of the cervix, gestational age, reason for labour induction, and Bishop score (Tables 1 and 2; Figure 1). Between the two groups, there was no significant difference in the initial questionnaire results in regard to valence, arousal, and virility. Similarly, there was no difference in the questionnaire parameters after delivery (Figure 2).

**Figure 3:**
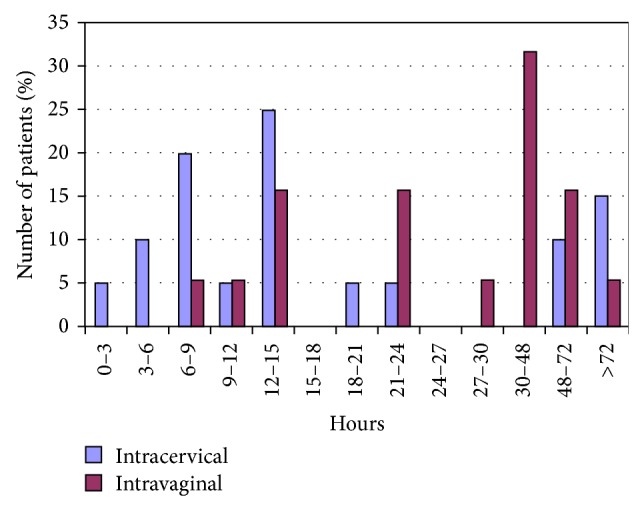
Induction-to-delivery time (h) of the two study groups.

Outcome parameters (Table 3) showed that the induction-to-delivery was statistically significantly shorter in the intracervical compared to the intravaginal group (median 12.8 h versus 29.9 h, *P* = 0.04; Figure 3). All other outcome parameters did not differ in the two groups (Table 3).

The major limitation of this study is the small sample group; therefore, the results of the study should be analysed with great caution.

## 4. Discussion

Our results show that intracervical PGE2 administration had a shorter induction-to-delivery time in comparison to intravaginal PGE2 without affecting the women's perception of the induction procedure. The quicker delivery rate with intracervical PGE2 is contrary to the current Cochrane database review, which included 11 trials [2, 12–22]. This difference might be explained that in the previous studies the cannula into the cervix was applied during a vaginal examination and not under direct visual control as in our study. This application technique during a vaginal examination is very difficult if the cervix is unripe (low Bishop score).

There is only one trial reported on maternal views, with three patients not satisfied in the intravaginal group (61 women) versus two patients in the intracervical group [5]. Our results have also shown no difference in the women's perception in regard to the application method.

Even though the statistical test did not show a significant difference in the delivery mode between the two groups, a difference of caesarean section rate of 25% (intracervical) versus 42% (intravaginal) would be interesting to evaluate in a bigger multicenter study. This difference can partly be explained due to difference in parity between the two groups (intracervical of median 2 versus intravaginal of median 1). Several studies identified parity as the most important factor influencing the successful vaginal delivery, whereas gestational age, fetal weight, and maternal BMI have much less impact [23–25]. Other studies have described no reduced risk of caesarean section [2, 5, 12–22, 26–39].

Similarly to previous studies [2, 13, 14, 17, 19, 26, 36, 37], FHR changes, which resulted in fetal blood sampling, were not different between the two methods of PGE2 administration.

## 5. Conclusion

Even though this small study is contrary to all other studies published so far, the intracervical PGE2 gel application should warrant more studies if the gel is applied under direct visual control. For the first time, intracervical PGE2 showed to be more effective than intravaginal prostaglandin. Even though the intracervical route is more difficult and uncomfortable for the woman, it does not seem to change the woman's perception of labour induction in comparison to the intravaginal route. Further larger multicentre studies are required to study the best route of administration.

## Figures and Tables

**Table 1:** Baseline characteristics of the study population of mean (M), ± standard deviation (SD), and median (n.s.: nonsignificant difference, *P* > 0.05).

	Cervical (*n* = 20)	Intravaginal (*n* = 19)	*P* value
	M ± SD (median)	M ± SD (median)	
Maternal age (years)	34.2 ± 4.5 (34)	32.5 ± 3.9 (32)	n.s.
Gravity	2.2 ± 1.3 (2)	1.8 ± 1.8 (1)	n.s.
Parity	0.7 ± 0.7 (1)	0.3 ± 0.6 (0)	n.s.
Gestational age (weeks)	39.6 ± 1.5 (40)	39.7 ± 1.1 (40)	n.s.
Bishop score	3.8 ± 1.3 (4)	3.5 ± 1.3 (3)	n.s.
Birth weight (kg)	3640 ± 401 (3685)	3556 ± 479 (3600)	n.s.
BMI (kg/m^2^)	23.8 ± 4.6 (23.1)	23.4 ± 3.4 (22.7)	n.s.

**Table 2:** Reason for induction of labour and outcome after induction of labour with either intracervical or intravaginal PGE2 (n.s.: nonsignificant difference, *P* > 0.05).

	Cervical (*n* = 20)	Intravaginal (*n* = 19)	*P* value
	*n* (%)	*n* (%)	
Reason for induction of labour			
Post date	6 (30)	7 (37)	n.s.
Premature rupture of membranes	4 (20)	5 (26)	
Preeclampsia	3 (15)	2 (11)	
On request	4 (20)	1 (5)	
Other	3 (15)	1 (5)	
Gestational diabetes	0	1 (5)	
IUGR	0	1 (5)	
Macrosomia	0	1 (5)	
Peridural anaesthesia	9 (45)	10 (53)	n.s.
Oxytocin augmentation	8 (40)	9 (47)	n.s.
Mode of delivery			
Vaginal birth (without operative delivery)	13 (65)	9 (47)	n.s.
Vacuum extraction/forceps	2 (10)	2 (11)	
Caesarean section	5 (25)	8 (42)	
Number of fetal blood sampling (%)			
0	14 (70)	15 (79)	n.s.
1	4 (20)	1 (5)	
2	1 (5)	3 (16)	
3	1 (5)	0	

**Table 3:** Outcome after induction of labour of mean (M), ± standard deviation (SD), and median (n.s.: nonsignificant difference, *P* > 0.05).

	Cervical (*n* = 20)	Intravaginal (*n* = 19)	*P* value
	M ± SD (median)	M ± SD (median)	
Arterial pH value	7.2 ± 0.08 (7.22)	7.23 ± 0.07 (7.22)	n.s.
5 min Apgar	9.8 ± 0.5 (10)	9.8 ± 0.1 (10)	n.s.
10 min Apgar	10 ± 0 (10)	10 ± 0.2 (10)	n.s.
Induction-to-delivery (hours)	38 ± 63 (12.8)	42 ± 12 (29.9)	0.04
Number of gel inductions	2.4 ± 2.2 (1)	2.0 ± 0.9 (2)	n.s.
